# How Magnetic Disturbance Influences the Attitude and Heading in Magnetic and Inertial Sensor-Based Orientation Estimation

**DOI:** 10.3390/s18010076

**Published:** 2017-12-28

**Authors:** Bingfei Fan, Qingguo Li, Tao Liu

**Affiliations:** 1 State Key Laboratory of Fluid Power and Mechatronic Systems, School of Mechanical Engineering, Zhejiang University, Hangzhou 310027, China; bingfeifan@foxmail.com; 2 Department of Mechanical and Materials Engineering, Queen’s University, Kingston, ON K7L 3N6, Canada; ql3@queensu.ca

**Keywords:** magnetic and inertial sensor, magnetic disturbance, attitude and heading decoupling, complementary filter, orientation estimation, Kalman filter

## Abstract

With the advancements in micro-electromechanical systems (MEMS) technologies, magnetic and inertial sensors are becoming more and more accurate, lightweight, smaller in size as well as low-cost, which in turn boosts their applications in human movement analysis. However, challenges still exist in the field of sensor orientation estimation, where magnetic disturbance represents one of the obstacles limiting their practical application. The objective of this paper is to systematically analyze exactly how magnetic disturbances affects the attitude and heading estimation for a magnetic and inertial sensor. First, we reviewed four major components dealing with magnetic disturbance, namely decoupling attitude estimation from magnetic reading, gyro bias estimation, adaptive strategies of compensating magnetic disturbance and sensor fusion algorithms. We review and analyze the features of existing methods of each component. Second, to understand each component in magnetic disturbance rejection, four representative sensor fusion methods were implemented, including gradient descent algorithms, improved explicit complementary filter, dual-linear Kalman filter and extended Kalman filter. Finally, a new standardized testing procedure has been developed to objectively assess the performance of each method against magnetic disturbance. Based upon the testing results, the strength and weakness of the existing sensor fusion methods were easily examined, and suggestions were presented for selecting a proper sensor fusion algorithm or developing new sensor fusion method.

## 1. Introduction

Advancements in micro-electromechanical systems (MEMS) technologies have made magnetic and inertial sensors more and more accurate, lightweight and low-cost, which has greatly promoted their usage in human motion analysis. These applications include walking speed estimation [1], gait analysis [2], pedestrian dead-reckoning (PDR) [3], activity classification [4], etc. Accurate sensor orientation is critical for these applications, and hence many studies endeavor to accurately estimate the sensor orientation [5,6,7,8,9]. Typically, a magnetic and inertial measurement unit (MIMU) is usually composed of a tri-axial accelerometer, a tri-axial gyroscope and a tri-axial magnetometer. The sensor orientation consists of roll, pitch and yaw in Euler angles representation, where roll and pitch are also called attitude, while yaw is called heading. In quasi-static condition and in a magnetically clean environment, the attitude of a sensor can be calculated from the measured gravitational acceleration, and the heading can be calculated from the measured geomagnetic field. Meanwhile, the attitude and heading can also be updated by angular velocity integration based on gyroscope measurements. However, each sensor has its own limitations, and these sensors may yield poor results when used alone due to the different characteristics of the magnetic and the inertial sensors [10]. On the one hand, accelerometers measure not only the gravitational acceleration, but also acceleration caused by motion. Hence, any motion of the sensor will cause an orientation estimation error. On the other hand, gyroscope-based orientation updates suffer from gyro integration drifts, and therefore the orientation error caused by integration tends to increase with time. Besides, magnetometer measurements are easily distorted by the so-called hard-iron and soft-iron magnetic distortions [11], which thereby affect the orientation estimation. To improve the orientation estimation accuracy, sensor fusion is necessary, where accelerometer and magnetometer measurements are used for compensating the drift during gyroscope data integration, and provide an absolute 3D orientation with respect to a common reference frame [5,12,13,14].

Accurate sensor orientation estimation is still a challenging task, and one of the limiting factors is the influence of magnetic disturbance on the magnetometer. In recent years, many researchers have focused on eliminating the negative effects of the magnetic disturbance [8,9,15,16]. De Vries et al. evaluated the magnetic distortion and its impact on the orientation estimation of a MIMU in a motion analysis labs [17], suggestions for eliminating its negative effects were given; Palermo et al. assessed the indoor magnetic distortion effects on gait analysis performed with wearable inertial sensors [18]; Robert-Lachaine et al. analyzed the effects of local magnetic field disturbances on the accuracy of magnetic and inertial sensors [19]. Commonly, these three studies analyze the effects of magnetic disturbance from the application perspective, where the sensor fusion methods were regarded as black boxes. Hence, the exact effects of the magnetic disturbance on the orientation of a given sensor fusion method were still unclear. Suh et al. proposed a quaternion-based indirect Kalman filter discarding pitch and roll information derived from magnetic sensors [15]. This work mainly focused on avoiding the influence of magnetic disturbance on pitch and roll estimations, no special treatment has been implemented to eliminate the negative effects on yaw estimation. Ligorio et al. presented a benchmarking procedure to assess the performance of sensor fusion methods [10], two Kalman-based sensor fusion algorithms were compared through the proposed procedure. The results recommended decoupling the attitude estimation from the magnetometer. However, the methods of eliminating the effect on yaw angle were not discussed. Bergamini et al. investigated the effectiveness of sensor fusion methods under different experimental conditions [20], with different time duration, presence/absence of static phases and etc. The representative stochastic (Extended Kalman Filter) and complementary (Non-linear observer) filters were analyzed. However, the magnitude or the dip angle of the magnetic disturbance were not presented in the results, and the magnetic disturbance in the test condition was not discussed quantitatively. Therefore, it is not clear about the exact influence of magnetic disturbance on the attitude and heading estimations. In short, these studies enable one to gain some information about magnetic disturbance, but still not in a systematic way. To better understand this problem and gain more insights, a comprehensive understanding of the existing methods on reducing the effect of magnetic disturbance is essential.

Recently, Ligorio and Sabatini reviewed the popular strategies to deal with the magnetic field disturbances in human motion analysis [21]. The authors summarized the magnetic-free attitude estimation methods, threshold-based approaches and model-based approaches for magnetic disturbance rejection. Through the experimental results of manual routine task and gait task test, the authors concluded that the model-based approach represented the best compromise. This review well summarized the popular approaches and analyzed the features of each method. However, some limitations exist in the review: (1) the magnetic disturbances in the experiments only contain short time (10 s) disturbance and periodic magnetic disturbance. As the disturbance conditions were not systematically designed, the weaknesses of a given algorithm may not be exposed. (2) The selected methods are the combinations the single-frame methods (TRIAD and QUEST) and magnetic disturbance rejection approaches (threshold-based and model-based), and they are all based on linear Kalman filter. However, other popular methods are not discussed, including gradient descent algorithm, improved explicit complimentary filter and extended Kalman Filter. Although the issues of magnetic disturbance become well known, no agreement on well-accepted strategies and the evaluation procedures has been reached. Therefore, a new up-to-date review on the effect of magnetic disturbance is necessary. In addition, in order to objectively assess the performance of MIMU in dealing with magnetic disturbance, a set of standardized evaluation procedures mimicking practical application scenarios are also essential.

The main contributions of this paper are summarized as follows: (1) we systematically review four major components of reducing the effects of magnetic disturbance on attitude and heading; (2) we propose a set of standardized testing methods for objectively evaluating the performance of sensor fusion method in a magnetically distorted environment; (3) we perform a comparison study with several well-known sensor fusion methods, and provide suggestions for selecting a proper sensor fusion algorithms (SFA) or developing new sensor fusion methods. The results of this work will lead to a better understanding of the effects of magnetic disturbance on the attitude and heading estimation. The rest of this paper is organized as follows: in Section 2, the major components dealing with magnetic disturbance are reviewed and analyzed. The standardized testing procedure and the aim of each test are introduced in Section 3. Results and discussion are provided in Section 4 and Section 5, respectively. Finally, in Section 6, conclusion and future work are presented.

## 2. Sensor Fusion Methods Based on Magnetic and Inertial Sensor

**Figure 1:**
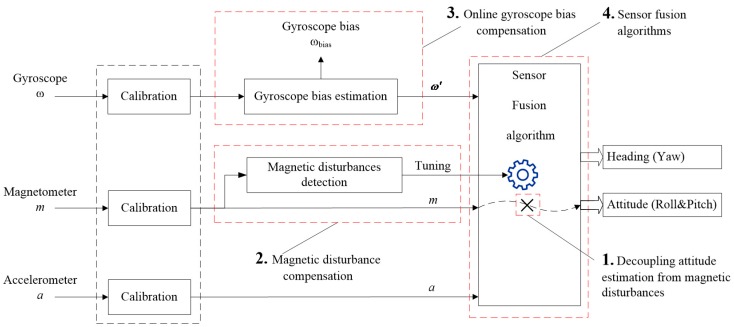
General procedure for orientation estimation based on MIMU. Four components were reviewed in the following subsections, including the methods of decoupling attitude estimation from magnetic disturbance, magnetic disturbance compensation, online gyroscope bias compensation and sensor fusion algorithms (SFAs).

For most sensor fusion methods, the block diagram can be summarized as shown in Figure 1. It can be seen that all the raw sensor data are calibrated first. Especially for a gyroscope, its calibration parameters consist of constant bias, scaling factor and bias instability [22]. The constant bias and scaling factor are determined during factory calibration, while the bias instability is removed by on-line gyro bias compensation. The calibrated magnetic field, acceleration and the compensated gyroscope data are fed to SFAs for sensor fusion. Meanwhile, the measured magnetic field is also used for magnetic disturbance detection, so the magnetic disturbance can be compensated through tuning the parameters contained in SFAs according to the detected result. In addition, in order to avoid the negative effect of magnetic disturbance on attitude, attitude estimation should be immune to magnetic disturbance, which has become an important part in SFAs.

In the last decade, the main work in this field focused on proposing new SFAs. The Kalman filter and complementary filter have become the most popular SFAs. In recent years, researchers are well aware that SFA is not the only important component in a MIMU when estimating orientation, because the SFA cannot be adapted to various ambient magnetic environments without the assistance of the other three components. This paper focuses on analyzing each of the four key components dealing with magnetic disturbance. The commonly-used methods and their features in each component are reviewed in the following subsections.

### 2.1. Decoupling Attitude Estimation from Magnetic Disturbance

Generally, an accelerometer and gyroscope are sufficient for attitude estimation. When magnetometer data is added in the orientation estimation, a new issue is introduced. For the example of the classic quaternion estimator (QUEST) [23], attitude estimation is also effected by magnetometer data. In this case, magnetic disturbance will not only directly affect the yaw accuracy, but also degrade the attitude estimation performance. Therefore, it is not desirable to use magnetic data in calculations related to pitch and roll [24,25]. The following are common methods used to decouple the attitude (pitch and roll) estimation from magnetic disturbance. Specially, the first two methods are used in the selected SFAs, while the other four methods are not directly used here but they are reviewed for completeness.

#### 2.1.1. TRi-Axial Attitude Determination (TRIAD) Algorithm

TRIAD algorithm is a classic method first presented in [23], and then has been widely used [10,21,26]. The TRIAD calculates the orientation of the global frame relative to the body frame. First, TRIAD uses two vectors to construct the orthonormal bases in the global frame and in the body frame as Equations (1) and (2):

(1) $$r_{1}=v_{1};\;\;\;\;r_{2}=\frac{r_{1}\times v_{2}}{\left|r_{1}\times v_{2}\right|};\;\;\;\;\;r_{3}=r_{1}\times r_{2}$$

(2) $$s_{1}=w_{1};\;\;\;\;s_{2}=\frac{s_{1}\times w_{2}}{\left|s_{1}\times w_{2}\right|};\;\;\;\;\;s_{3}=s_{1}\times s_{2}$$

where $v_{1}\;\mathrm{and}\;v_{2}$ are two nonparallel reference unit vectors in the global frame; $w_{1}\;\mathrm{and}\;w_{2}$ are the corresponding observation normalized vectors measured in the body frame.

These triads are then used to create measurement and reference matrices. The orientation of the global frame relative to the body frame A can be calculated by Equation (3):

(3) $$\{\begin{array}{c} M_{\mathrm{ref}}\;\;=\left[r_{1}\;\;r_{2}\;\;r_{3}\right]\\ M_{\mathrm{meas}}=\left[s_{1}\;\;s_{2}\;\;s_{3}\right] \end{array}\;\;\;\;\;\;\;\;\to \;\;\;\;\;\;\;\;\;\;\;\;\;\;A=\;M_{\mathrm{meas}}M_{\mathrm{ref}}^{T}$$

In the TRIAD algorithm, the first vector is the dominant vector. Therefore, when using the TRIAD algorithm to calculate the orientation, the more reliable acceleration is used as the first vector, while the problematic magnetometer data is used as the second vector. The reference vectors can be expressed as:

$$v_{1}=g,\;v_{2}=m_{loc}$$

$$r_{1}=g=\left[\begin{array}{c} 0\\ 0\\ 1 \end{array}\right];r_{2}=\left[\begin{array}{c} 0\\ 1\\ 0 \end{array}\right];r_{3}=\left[\begin{array}{c} 1\\ 0\\ 0 \end{array}\right]$$

$$s_{1}=\left[\begin{array}{c} x_{11}\\ x_{21}\\ x_{31} \end{array}\right];s_{2}=\left[\begin{array}{c} x_{12}\\ x_{22}\\ x_{32} \end{array}\right];s_{3}=\left[\begin{array}{c} x_{13}\\ x_{23}\\ x_{33} \end{array}\right]$$

where g is the gravitational acceleration and $m_{loc}$ is the local geomagnetic field; $s_{1}$ denotes the normalized acceleration measured in the body frame; $s_{2}\;\mathrm{and}\;s_{3}$ are the vectors calculated using Equation (2). 

Hence, the calculated orientation $A=\;\left[\begin{array}{ccc} x_{13} & x_{12} & x_{11}\\ x_{23} & x_{22} & x_{21}\\ x_{33} & x_{32} & x_{31} \end{array}\right]$ according to Equation (3). Then, the Euler angles can be calculated as Equation (4):

(4) $$\mathrm{Roll}\;\phi =\mathrm{atan}2\left(x_{21},x_{31}\right)\;\mathrm{Pitch}\;\theta =-\mathrm{asin}\left(x_{11}\right)\;\mathrm{Yaw}\;\psi =\mathrm{atan}2\left(x_{12},x_{13}\right)$$

It can be seen that pitch and roll are determined only by the components (bold font) related to gravitational acceleration, $s_{1}$, and therefore they are immune to magnetic disturbance.

#### 2.1.2. Construct A New Reference Vector Instead of Geomagnetic Field

**Figure 2:**
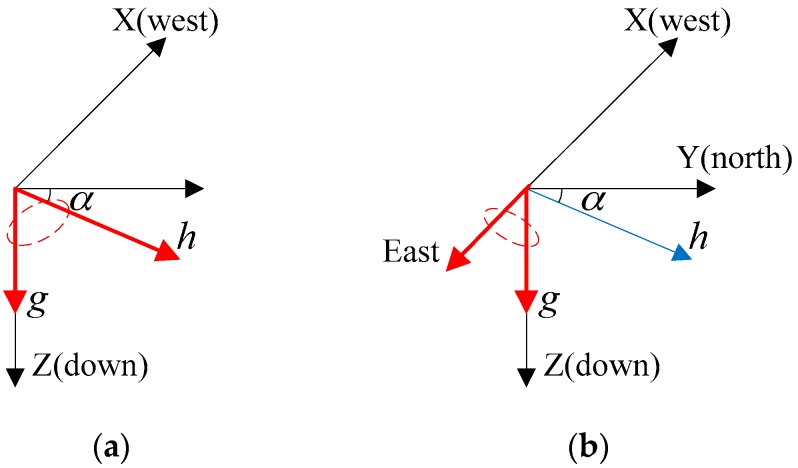
The decoupling method of using a new reference vector instead of geomagnetic field [27]. (**a**) Legacy reference vectors (red arrows) are gravitational acceleration and geomagnetic field, (**b**) New reference vectors are gravitational acceleration and East vector (red arrows).

Decoupling of input signals of accelerometer and magnetometer is an innovative method that prevents the attitude estimation from the influence of magnetic disturbance. Martin and Salaun proposed a simple solution by creating another inertial vector as the cross product of the gravitational acceleration, g, and the Earth’s magnetic field, h [25,27,28]. The corresponding “virtual” measurement is based on the cross product of accelerometer, a, and magnetometer measurements, m. More specifically, the following vectors are calculated, using virtual East$\;V_{E}=\frac{g\times h}{\left|g\times h\right|}$ as the reference vector instead of the Earth’s magnetic field, and using $V_{E\_mes}=\frac{a\times m}{\left|a\times m\right|}$ as the corresponding measurement vector instead of magnetometer measurements. The constructed new reference vectors are also shown in Figure 2.

This method decouples attitude and heading estimation from the input signals of accelerometer and magnetometer, and it does not require any changes in the SFA when applied to explicit complementary filter [25]. This is a simple and effective solution to make attitude immune to magnetic disturbance with no need for complex algebraic derivation.

#### 2.1.3. Other Commonly-Used Decoupling Methods

(a) Two-Step Orientation Estimation

**Figure 3:**
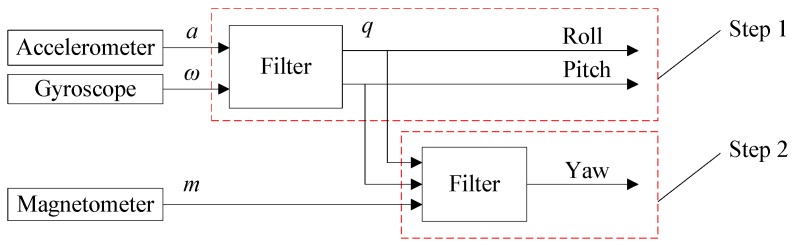
Block diagram of the general concepts of most two-step orientation estimation methods.

There are several two-step orientation estimation implementations [15,29,30,31]. The general concepts of most existing two-step methods are similar. Figure 3 shows the block diagram of a typical two-step orientation estimation method [29]. The method breaks the measurement update process into two steps: (1) estimates pitch and roll using acceleration and angular velocity; (2) estimates yaw angle with the estimated pitch, roll and the magnetometer data. Apparently, pitch and roll estimations have no relationship with magnetometer reading.

The main advantage of this method is that it does not require too much complex algebraic derivation and modification of the basic sensor fusion algorithms. Both Kalman filter and complementary filter based SFAs can be adopted to achieve two-step orientation estimation, but it may be unnecessary to use this method if one-step estimation method can achieve the feature, that attitude estimation is immune to magnetic disturbance.

(b) Factored Quaternion Algorithm 

Factored quaternion algorithm (FQA) is an algebraic solution to orientation calculation based on quaternion [24]. This method divides quaternion into three components: roll quaternion, pitch quaternion and yaw quaternion. Each quaternion is described as follows:

Roll quaternion:

$$q_{r}=\cos\frac{\phi }{2}\left(1\;\;0\;\;0\;\;0\right)+\sin\frac{\phi }{2}\left(0\;\;1\;\;0\;\;0\right)$$

Pitch quaternion:

$$q_{p}=\cos\frac{\theta }{2}\left(1\;\;0\;\;0\;\;0\right)+\sin\frac{\theta }{2}\left(0\;\;0\;\;1\;\;0\right)$$

Yaw quaternion:

$$q_{y}=\cos\frac{\varphi }{2}\left(1\;\;0\;\;0\;\;0\right)+\sin\frac{\varphi }{2}\left(0\;\;0\;\;0\;\;1\right)$$

Finally, the orientation is calculated through quaternion multiplication:

$$\hat{q}=q_{y}⊗q_{p}⊗q_{r}$$

Through a series of algebraic derivation, $q_{r}$ and $q_{p}$ can be expressed by equations of the measured acceleration. Hence, pitch and roll estimations are immune to magnetic disturbance. FQA is a single frame approach, and it can be integrated into an complementary filter method [32].

(c) Algebraic Quaternion Algorithm

Valenti et al. proposed an algebraic quaternion method similar to FQA [6,33]. This method divides quaternion into two components: accelerometer quaternion $q_{acc}$ and magnetometer quaternion $q_{mag}$. The orientation can be calculated by Equation (5). The method can also be used in sensor fusion with gyroscope, as described in Equation (6):

(5) qGB=qacc⊗qmag

(6) qGB  =qGBω⊗ Δq^acc⊗Δq^mag

where qGB  denotes the orientation of the global frame relative to the body frame. qGBω is the gyroscope update component. $q_{acc},\;{\hat{\Delta q}}_{acc}$ and $q_{mag},\;\;{\hat{\Delta q}}_{mag}$ are the corrections according to the accelerometer and magnetometer measurements, respectively.

$q_{acc}$ and $\;{\hat{\Delta q}}_{acc}$ are the derived expression from measured acceleration. $q_{mag}$ and ${\hat{\Delta q}}_{mag}$ have only a single degree of freedom: $q_{mag}=\left[\begin{array}{cccc} q_{0mag} & 0 & 0 & q_{3mag} \end{array}\right]$, such that the quaternion multiplication will not influence the pitch and roll. Through this method, pitch and roll estimations are immune to magnetic disturbance. 

(d) Arc-Tangent Attitude Solution

Arc-tangent attitude solution [34] is a method that calculates Euler angles directly, as described by Equations (7) and (8):

(7) $$\theta =-\arctan\left(\frac{g_{x}^{b}}{\sqrt{{\left(g_{x}^{b}\right)}^{2}+{\left(g_{z}^{b}\right)}^{2}}}\right),\;\phi =\arctan\left(\frac{g_{y}^{b}}{g_{z}^{b}}\right)$$

(8) $$\psi =\arctan\left(\frac{-m_{y}^{b}\cos\;\phi +m_{z}^{b}\sin\;\phi }{m_{x}^{b}\cos\;\theta +m_{y}^{b}\sin\;\phi \sin\;\theta +m_{z}^{b}\cos\;\phi \sin\;\theta }\right)$$

where $g_{x}^{b}$, $g_{y}^{b}$ and $g_{z}^{b}$ denote the X, Y and Z components of the measured acceleration. $m_{x}^{b},{\;m}_{y}^{b}{\;\mathrm{and}\;m}_{z}^{b}$ represent the X, Y and Z components of the measured magnetic field.

From the Equation (7), it can be seen that pitch and roll are determined only by the measured acceleration. Hence, they are immune to magnetic disturbance. The disadvantage of this method is that the Euler angle’s singularity problem will happen when the pitch is close to ±90°. 

### 2.2. Magnetic Disturbance Compensation

In sensor fusion algorithms, the measured magnetic field is used for yaw correction. When the magnetic field is distorted by ambient ferromagnetic objects, heading errors will inevitably be introduced, but fortunately, headings can also be updated by gyroscope data integration, which is not influenced by magnetic disturbances. Through exploring the potential of gyroscope, the effect of magnetic disturbances can be suppressed. By reviewing the scientific literature, the methods of reducing the influence of magnetic disturbance can be divided into three categories: threshold-based methods, model-based methods and the combination of both methods.

#### 2.2.1. Threshold-Based Methods

Threshold-based methods are also called vector selection methods [35,36]. A typical threshold method contains two parts: magnetic disturbance detection and rejection. The detection is the precondition of magnetic disturbance rejection. When the geomagnetic field is distorted by ambient ferromagnetic objects, the magnitude and dip angle would vary drastically. Based on this principle, the magnetic disturbance can be detected by setting thresholds of magnitude, dip angle or both of them. The detection can use the instantaneous value [35,36,37] or the covariance of current magnetic field [7]. Detection by covariance is considered more stable than that by instantaneous value but requires extra computation. 

More precisely, the principle of threshold-based methods can be described as Equation (9), where m is the magnetic field date that is fed to SFA. When detected magnetic disturbance, using predicted magnetic field in place of measured value to reduce error caused by incorrect reference magnetic field:

(9) $$m=\{\begin{array}{c} \;\;m_{measured},\;\;\;\;\mathrm{no}\;\mathrm{magnetic}\;\mathrm{disturbance}\;\mathrm{detected}\;\\ \;\;m_{predicted},\;\;\;\;\;\mathrm{magnetic}\;\mathrm{disturbance}\;\mathrm{detected}\; \end{array}$$

Threshold-based methods can also be used to adaptively set the measurement covariance in Kalman filter-based methods [5]. As described in Equation (10), when the magnetic disturbance are detected, the magnetic noise σ Rh2 would be set to infinite, such that the SFA would not trust the magnetometer measurement. This strategy, also called R-adaption, belongs to the class of noise adaptive approaches [38]:

(10) σ Rh2={σh2,                             normal state∞,                         disturbed state

Threshold-based methods are easy to implement and do not require too much extra computation. However, the threshold is usually not easy to set and the behavior of the algorithm can be somewhat unstable for values close to the set threshold value [21]. Although this problem can be eliminated by setting two thresholds [6], it is hard to select a perfect threshold.

#### 2.2.2. Model-Based Methods

Generally, model-based methods are based on the assumption that the magnetic disturbance obeys a specific model and attempts to augment the state vectors in the SFA by adding the magnetic disturbance, such that the magnetic disturbance can be estimated at the same time as the orientation. Roetenberg et al. proposed a complementary Kalman filter to estimate sensor orientation [9]. In the filter, the magnetic disturbance was modeled as a first order Markov process, and was estimated with the orientation. Sabatini et al. modeled the magnetic disturbance as a first-order Gauss-Markov stochastic process [26], and then compensated magnetic disturbance using extended Kalman filter. In these ways, the magnetic data does not have to compare with any threshold. The model-based compensation methods avoid the problem of tuning appropriate thresholds. However, when the external disturbance does not obey the proposed sensor model, large errors will be introduced. In practice, it is hard to propose a model that can be adapted to complex ambient magnetic disturbance.

#### 2.2.3. The Combination of Both Threshold-Based Methods and Model-Based Methods

In human motion analysis with magnetic and inertial sensors, the ambient magnetic disturbance is sophisticated. Neither threshold-based methods nor model-based methods can conquer the complex ambient magnetic disturbance alone. In practical applications, sensor fusion method usually combines the model-based method and the threshold-based method, which represents a new research direction. Sabatini et al. proposed a variable-state-dimension extended Kalman filter (VSD-EKF) for estimating the 3D orientation [39]. This SFA has two models for the magnetic disturbance, one for small disturbance and the other one for large disturbance. The switching rule is based on threshold-based method. Tian et al. proposed an adaptive-gain complementary filter for real-time human motion tracking [8], in which the threshold-based method is also used as a sub-method. The combination of these two methods can potentially improve the accuracy compared to individual method. 

### 2.3. On-line Gyro Bias Estimation

The accuracy of a gyroscope directly affects the accuracy of yaw estimation, especially in a magnetically distorted environment. In this situation, the magnetic reference vector misses, and the yaw estimation mainly depends on the gyroscope data integration. Suppose that the gyro bias is 0.1°/s, the numerical integration error of angle will increase 6° in every 60 s, causing a significant error. Hence, in an environment with magnetic disturbance, the accuracy of gyroscope is crucial, and therefore the gyro bias needs to be removed. Several studies have concentrated on gyro bias estimation and the commonly-used methods include no-motion gyro bias update method, model-based gyro bias estimation method and low pass filter method.

#### 2.3.1. No-Motion Gyro Bias Update Method

The no-motion gyro bias update method is a simple and valid method for gyro bias estimation. This approach takes the average outputs of the gyro as the bias when the sensor is detected in a static state or moving at constant velocity without rotation [26], and then the bias-free angular velocity can be obtained by subtracting the bias. This method is only valid when the motion of the sensor contains static state. The disadvantage of this method is that it might omit small amplitude signals in static state and lead to undesirable step changes of the gyro bias between each estimation [40].

#### 2.3.2. Model-Based Gyro Bias Update Method

This method does not estimate the gyro bias separately, but rather includes the gyro bias into the state vector, thus estimating the gyro bias and the orientation simultaneously. Zhang et al. presented an EKF to estimate the orientation and gyro bias [41]. The state vector includes not only the quaternion, but also the gyro bias. Roetenberg et al. proposed a complementary Kalman filter based on an error model, the gyro bias error was also included in the model states [9]. In this way, the gyro bias error was estimated the same time as the orientation. 

The stability of the gyro bias is quantified as the minimum Allan variance [8]. It is a slow-changing signal. Including the gyro bias into the state vector will increase the computational load of orientation estimation. Actually, gyro bias does not need to be updated at such a high frequency as the orientation. 

#### 2.3.3. Low-Pass Filter Gyro Bias Update Method

In order to overcome the shortcomings of the no-motion gyro bias update method, and considering that the gyro bias is a slow-varying signal, the gyro bias has been considered as a low frequency noise and estimated by using a low-pass filter [6,40]. The low-pass filter is applied only when the sensor is in a steady-state condition, avoiding filtering useful information during dynamic movement. The low-pass filter can be described as Equations (11) and (12):

(11) $$\omega_{bias}=2\pi f_{c}\int p\cdot \omega \;dt$$

(12) $$p=\{\begin{array}{c} 1\;\;\;\;\mathrm{if}\;f_{b}\left(\omega ,\omega_{min}\right)>t_{b}\\ 0\;\;\;\;\mathrm{else}\; \end{array}$$

where $\omega_{bias}$ is the gyro bias; $f_{c}$ is the corner frequency; *p* is static condition; the function $f_{b}\left(\omega ,\omega_{min}\right)$ computes the time when the magnitude of each element of ω has been below $\omega_{min}$. $t_{b}$ is the minimum static period; ω represents the angular velocity; $\omega_{min}$ is the threshold of steady-state condition.

Compared with the no-motion gyro bias update method, the low-pass filter gyro bias update approach reduces the risk of corrupting small amplitude signals and undesirable step changes of the gyro bias. Moreover, it requires less computation compared with a model-based method.

### 2.4. Selected Representative Sensor Fusion Algorithms (SFAs)

SFA is the core of any sensor fusion method. Researchers have proposed many different SFAs, such as linear Kalman filter, unscented Kalman filter, extended Kalman filter, gradient descent algorithms and explicit complementary filter. In order to enable one to have a better understanding of current well-known SFAs, especially their performance in a magnetically distorted environment, we performed a comparison study with four representative SFAs. The selected SFAs are dual-linear Kalman filter with TRIAD algorithm (DLKF), extended Kalman filter with gyro bias estimation (EKF), gradient descent algorithm (GDA), and the improved explicit complementary filter (IECF). All the selected SFAs are based on quaternion, because the quaternion is arguably the most popular orientation representation and does not exhibit the singularity problem. It can be easily converted to Euler angles and rotation matrix. Euler angle is also used in this paper but mainly for visualization, and its sequence is ‘ZYX’ order. 

#### 2.4.1. Dual-Linear Kalman Filter with TRIAD Algorithm (DLKF)

**Figure 4:**
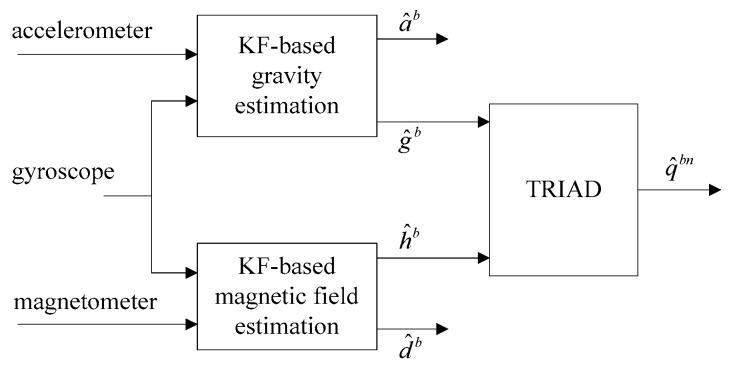
Overview of dual-linear Kalman Filter with TRIAD algorithm [42]. One Kalman filter separates the Earth’s gravitational ${\hat{g}}^{b}$ and the external acceleration ${\hat{a}}^{b}$. The other one separates the geomagnetic field ${\hat{h}}^{b}$ and the magnetic disturbance ${\hat{d}}^{b}$.

Ligorio et al. proposed a linear Kalman filter method for inertial measurement units (IMUs) [42], and then extended it to MIMU [21]. As shown in Figure 4, DLKF is composed of two parallel linear Kalman filters. The estimated Earth’s gravitational and geomagnetic field are then used to calculate the orientation ${\hat{q}}^{bn}$ using TRIAD algorithm. This method is a combination of stochastic approach and deterministic approach. The computational cost of this method is lower than a single extended Kalman filter. Because of the working principle of the TRIAD algorithm, pitch and roll estimations are decoupled from the magnetic reading.

#### 2.4.2. Extended Kalman Filter with Gyro Bias Estimation (EKF)

The extended Kalman Filter method is defined as a set of standard Kalman Filter equations, and it can be simply redefined for various models through the reconstruction of state vector. The EKF estimating the quaternion $q_{0}-q_{3}$ , and gyro bias $\;\;b_{\omega x}-\;\;b_{\omega z}$ is a commonly-used SFA [41,43,44], and its state vector is described as Equation (13):

(13) $$x_{1\times 7}=\left[q_{0}\;\;\;q_{1}\;\;\;q_{2}\;\;\;q_{3}\;\;\;b_{\omega x}\;\;b_{\omega y}\;\;b_{\omega z}\right]$$

The EKF method with state vector which contains gyro bias can estimate the gyro bias in real time. Compared with the no-motion gyro bias update and low-pass filter methods, this gyro bias estimation method does not require static phases, but increases the computational load. Moreover, because of the principle of EKF [6], pitch and roll estimations are not decoupled from magnetic disturbances. Another disadvantage of EKF is that it requires to predefine the reference direction of the Earth’s magnetic field [12], and the inaccuracy of the definition will cause error to yaw angle as well as pitch and roll angles.

#### 2.4.3. Gradient Descent Algorithm (GDA)

Madgwick et al. proposed a gradient descent algorithm for orientation estimation based on IMU and MIMU [12], and this algorithm invoked extensive attentions. Many researchers use GDA as a basic algorithm to propose further improvement [37,45] , or as a comparison algorithm to show the merits of their proposed algorithms [6,7,31]. Indeed, GDA has several advantages, such as low computational cost, no need for defining the reference direction of the earth’s magnetic field and easy tuning. But there are also some well-known shortcomings, such as the pitch and roll estimation are not decoupled from magnetic disturbances [6,31,46], and it contains only one adjustable parameter, which made it hard to find a perfect value for both accelerometer and magnetometer, as the characteristics of them are different.

#### 2.4.4. Improved Explicit Complementary Filter (IECF)

Actually, Madgwick regarded the gradient descent algorithm (GDA) [12] as a preliminary work of his study. An improved method is presented in his later studies, and it was proved to have better performance [40]. In this paper, we called it improved explicit complementary filter (IECF). The IECF is designed based on the scheme of Mahony’s explicit complementary filter [13,47]. It is an open source algorithms that is employed by a commercially available products x-IMU [48], and has been used by many users [40]. 

The IECF can be simply described as Equations (14) and (15). qEI describes the orientation of the earth relative to the IMU, and it is obtained through the integration of the rate of change of the quaternion. q˙EI is computed by Equation (15), and it is a function of normalized estimated quaternion, q^EI, the gyroscope measurement $\vec{\omega }$ and an error term, $\vec{e}$, scaled by the algorithm gain K:

(14) qEI=∫q˙EIdt

(15) q˙EI=12q^EI⊗[ 0  (ω→+Ke→)T ]

(16) $$\vec{e}=\;{\vec{e}}_{a}+{\vec{e}}_{m}$$

The error $\vec{e}$ is determined from the accelerometer and magnetometer measurements with each sensor providing a separate error component. ${\vec{e}}_{a}$ is calculated as the cross product of the normalized measured acceleration and the predict acceleration. While ${\vec{e}}_{m}$ is calculated as the cross product of the normalized measured east and predict east, as described in Section 2.1.2. In this way, pitch and roll estimations are decoupled from magnetic reading.

**Figure 5:**
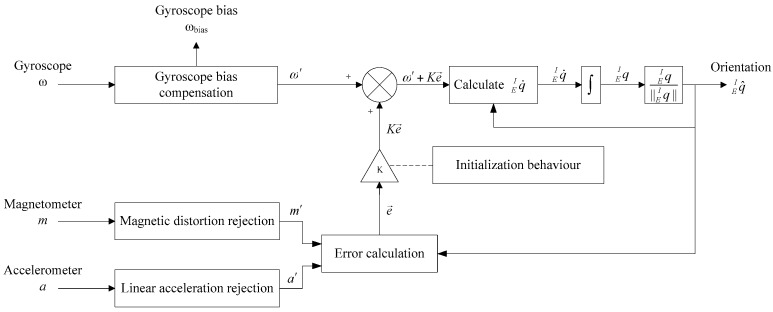
Block diagram of the improved explicit complementary filter (IECF).

The block diagram of IECF is shown in Figure 5. In summary, the IECF has following improvements compared with the GDA:

1. Pitch and roll estimations are decoupled from magnetic disturbance.
2. Fast initialization behavior

In the initialization period, the algorithm gain K was set to a large value for fast convergence. After initialization, K was set to the smaller normal value. In this way, the initialization time can be reduced.

3. Gyro bias compensation

Gyro bias estimation is considered as an essential part of sensor fusion. IECF performs the gyro bias compensation by the low-pass filter method, as described in Section 2.3.3. Gyro bias was estimated in a steady condition and then subtracted from the angular velocity.

4. Magnetic distortion rejection

IECF simply validates the magnetic field by setting a threshold range for the magnetometer, any value outside this range will be discarded. 

#### 2.4.5. IECF Use only Acceleration and Gyroscope Data (IECF_6_)

The block diagram of IECF_6_ is similar to that of IECF. The only difference is that IECF uses nine-axis data from accelerometer, gyroscope and magnetometer, while the IECF_6_ uses only six-axis data discarding the magnetic information. The IECF_6_ is intended to be used in a seriously magnetically distorted condition and provides a reference yaw estimation based on the integration of angular velocity. In theory, a good adaptive sensor algorithm should perform as well as IECF_6_ in a critical magnetically distorted environment but still provide absolute yaw angle.

## 3. Experimental Setting

In this section, we evaluated the performance of SFAs under different testing conditions. The SFAs were tested using the data collected by a commercially available MIMU, i.e., XSENS MTw (XSENS B.V. Technologies, Enschede, Netherlands) which includes tri-axis accelerometers, gyroscopes, and magnetometers. All the sensors were calibrated before they were delivered, and the magnetometer was recalibrated in the field with its accompanying tool. The raw calibrated sensor data was logged to PC wirelessly at 100 Hz. According to the recommendation in the user manual [49], the MTw was initialized in a magnetically clean environment and warmed up for several minutes before data collection. In order to ensure sufficient convergence time for the selected off-line SFAs, each dataset contains 20–30 s of data under static condition.

**Figure 6:**
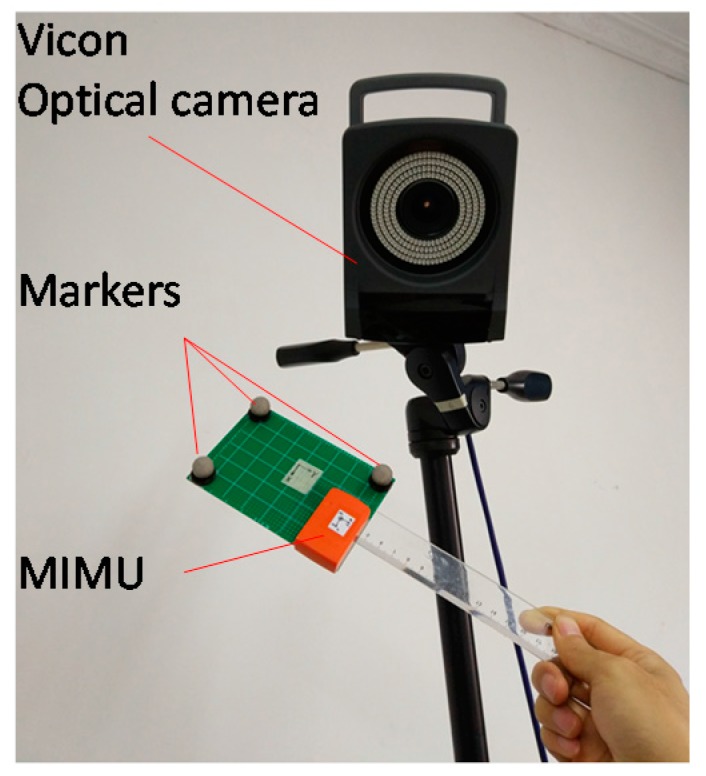
A rigid-body object where both makers and MIMU are attached.

A six-camera optical motion capture system (Vicon T40s, Oxford, UK) served as the gold standard. As shown in Figure 6, the sensor was attached on a rigid body with three makers. The ground-truth orientation was calculated through the Gram-Schmidt orthogonalization procedure. Both the sample rate of the MIMU and Vicon were set at 100 Hz, and the two systems were time-synchronized manually. The alignment error between the optical motion capture system and MIMU system was compensated using the well-accepted method introduced in [20,50]. The principle of this method is to calculate the angular velocities of both devices during an alignment procedure, and then calculate the alignment quaternion between both local frames using an optimization algorithm. The alignment procedure consists of rotations around three more or less orthogonal axes, which can be performed by hands easily. The quaternion estimated by MIMU was multiplied by the alignment quaternion when compared with the quaternion of the optical motion capture.

For all the SFAs except the EKF, the gyro bias is removed through the low pass filter method. The parameters of the low pass filter are $f_{c}=0.05$ Hz; $\omega_{min}=3{}^{\circ}/s$; $t_{b}=2\;s$. The removal of gyro bias is unnecessary for EKF because it has this function. Besides, the parameters of the selected SFAs are set according to the recommend methods introduced in the original literature. The detailed settings for each sensor fusion algorithms are listed in Table 1.

In order to objectively assess the performance of each algorithm, we intentionally designed five standardized tests, which includes general static, dynamic tests and special tests in different magnetic environments (Table 2). The objective of each test is listed in the second column, which covers the important factors related to the performance in magnetically distorted environments. 

**Figure 7:**
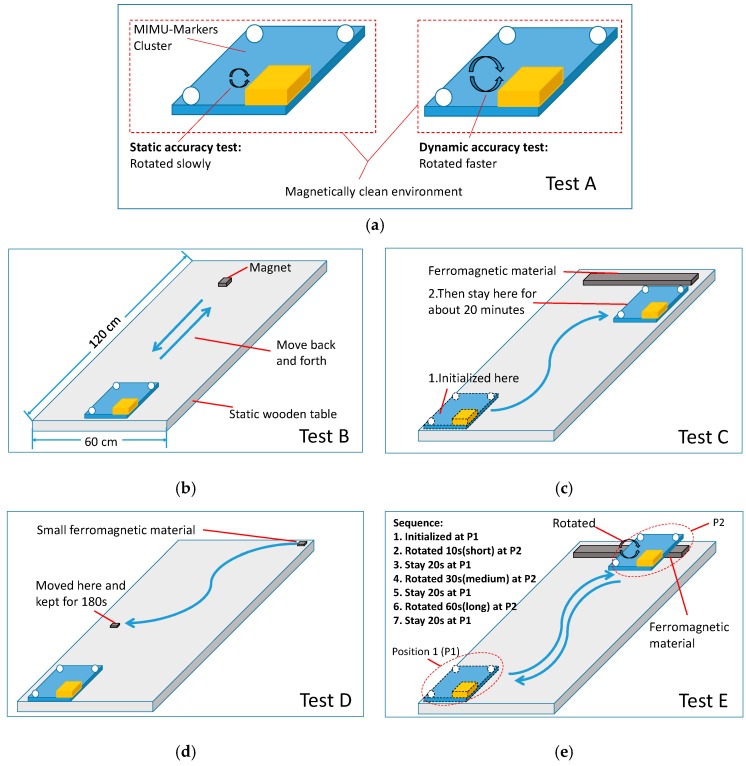
The test scenarios for the proposed standardized tests. (**a**) Test A: Static and dynamic accuracy without magnetic disturbance; (**b**) Test B: Pitch and roll estimations with magnetic disturbance; (**c**) Test C: Long-time stability of yaw angle with continuous magnetic disturbance; (**d**) Test D: Yaw estimation with small magnetic disturbance in static condition; (**e**) Test E: Dynamic accuracy with continuous magnetic disturbance.

The test scenarios for the proposed standardized tests are shown in Figure 7, and the detailed experimental protocols are described in the following subsections. In these tests, the magnetically clean environment denotes an indoor place at least 40 cm away from any magnetic sources [17], and the variation of the magnitude of magnetic field measured by the MIMU is within 2%. The magnitude and dip angle of the magnetic field are plotted to show the severity of the magnetic disturbance, as they are commonly-used indices [7]. The magnitude is the norm of the magnetometer measurement, and the dip angle $\theta_{dip}$ is calculated by Equation (17):

(17) $$\theta_{dip}=\frac{\pi }{2}-\arccos\left(\left(A\left(q\right)h\right)\cdot g/∥h∥\right)$$

where A(q) is the rotation matrix converted from the estimated orientation of sensor. g is the normalized gravity acceleration, and h is the measured magnetic field.

### 3.1. Test A: Static and Dynamic Accuracy without Magnetic Disturbance

Static and dynamic accuracy in a magnetically clean environment are the basic specification when evaluating a SFA. Hence, we performed these general tests first. In this scenario, as shown in Figure 7a, the MIMU was put in a magnetically clean environment, which was pre-checked by the same MIMU through verifying the measured magnetic field. During the test, the MIMU was rotated manually for about 60 s and each trial was repeated five times. The collected data was fed to different sensor fusion algorithms. The static and dynamic RMSEs of estimated Euler angles and its standard deviations were calculated and compared, with assuming a static condition when the angular velocity measured by the MIMU was <20°/s, and a dynamic condition when ≥ 20°/s. This assumption enabled the MIMU collect more data in static accuracy test. In addition, to demonstrate the importance of the gyro bias removal, we performed orientation estimation using integration method with one of the dynamic dataset. The estimated orientations are compared with the values obtained from the optical motion capture.

### 3.2. Test B: Pitch and Roll Estimations with Magnetic Disturbance

Whether pitch and roll estimations are decoupled from magnetic disturbance is an important feature for a SFA. In this scenario, as shown in Figure 7b, the MIMU was kept static in a magnetically clean environment, and then a small magnet was moved closer to the MIMU five times to create a magnetic disturbance. This test lasted about 25 s. The estimated Euler angles of different SFAs were compared directly, to check whether pitch and roll estimations were immune to magnetic disturbance. The maximum deviations of Euler angles were calculated with a comparison to the initial values to quantify the influence the magnetic disturbance. Note that all the adaptive strategies for magnetic disturbance compensation were disabled for assessing the features of the basic sensor fusion algorithms. 

### 3.3. Test C: Long-Time Stability of Yaw Angle with Continuous Magnetic Disturbance

For human motion analysis with MIMU, the sensor is usually strapped to a body segment and the segments might be moved to a magnetically distorted environment and placed for a while. This will cause long-duration magnetic disturbance. In this situation, the reference vector of yaw angle is distorted. A common strategy is to discard magnetic reading and to estimate yaw angle by numerical integration of angular velocity. In order to quantify the estimation error and check if the gyro bias was removed properly, we designed this test. In this test, as shown in Figure 7c, the MIMU was initialized in a magnetically clean environment, and then was moved closer to a ferromagnetic material and stayed for about 20 min to mimic long-duration magnetically distorted environment. The deviations of the estimated yaw angles of the SFAs were compared accordingly.

### 3.4. Test D: Yaw Estimation with Small Magnetic Disturbance in Static Condition

Generally, in our daily-life surroundings, as long as the MIMU is not moved closer to the magnetic disturbance source deliberately, it more likely encounters an environment with small magnetic disturbance. For threshold-based method, it is hard to cope with small magnetic disturbance below the threshold. This distorted magnetic field will be regard as good samples by the algorithm and potentially lead to big yaw error. Thus, creating a small magnetic disturbance scenario can test if a given algorithm can deal with this case properly. In this test, as shown in Figure 7d, the MIMU was put static on the table, and then a small ferromagnetic material was moved closer to the MIMU and stayed for about 2 min. Meanwhile, we made sure that the amplitude of the disturbance was of small value (less than the threshold) through the observation of the measured magnetic field. The collected sensor data was fed to all the selected SFAs; the estimated yaw angles were compared directly, and the convergence behaviors of different SFAs were analyzed.

### 3.5. Test E: Dynamic Accuracy with Continuous Magnetic Disturbance

When we fix a MIMU on a foot to perform gait analysis, the MIMU might be close to ferromagnetic materials contained in the floor of our building, which creates a dynamic condition with continuous magnetic disturbance. Hence, it is essential to test the sensor in this critical condition. For MEMS based gyroscopes, the measurement error grows with the increase of angular velocity. In static condition with continuous magnetic disturbance, as long as the gyro bias was removed properly, yaw angle estimation based on numerical angular velocity integration would not diverge rapidly, but in dynamic conditions with continuous magnetic disturbances, accurate yaw estimation not only requires one to remove gyro bias, but also requires accurate angular velocity measurements. Hence, this test represents the most critical condition for any magnetic disturbance compensation strategies. Moreover, in order to analyze the relationship between error and duration, we created continuous magnetic disturbance with different durations. During the test, as shown in Figure 7e, there are two positions, where P1 denotes the magnetically clean environment and P2 represents magnetically distorted environment. The action sequence is listed in the Figure 7e. The MIMU was first initialized at P1, then was moved to P2 and rotated for a certain period, and then was placed at P1 for 20 s. The duration of rotations are 10, 30 and 60 s, respectively. In this way, the short, medium and long magnetic disturbance were mimicked. The collected data was fed to all the selected fusion algorithms. All the SFAs adopted the threshold-based magnetic disturbance rejection method, and the magnitude thresholds were set as 0.05 a.u. This test was repeated five times, and the RMSEs and standard deviations of the Euler angles of different durations were calculated respectively.

## 4. Results

### 4.1. Test A: Static and Dynamic Accuracy without Magnetic Disturbance

The RMSEs of Euler angles of different SFAs are listed in Table 3. As can be seen from the results, in static condition, the accuracy of different SFAs are similar, and all the SFAs achieve a high accuracy. However, in dynamic condition, the accuracy of different SFAs varies, where the performance of IECF and DLKF is better than that of EKF and GDA.

**Figure 8:**
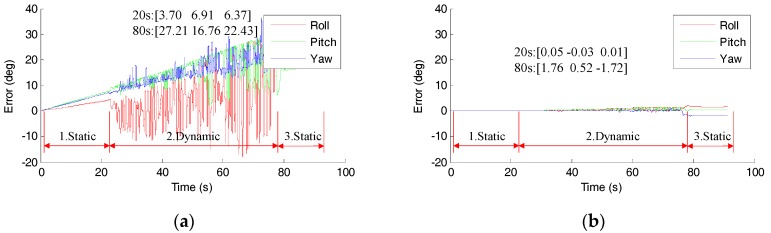
The error of orientation estimation using integration method. (**a**) Without the removal of gyro bias; (**b**) with the removal of gyro bias.

Figure 8 shows the errors of the estimated orientation using only integration method with and without the removal of gyro bias, by enabling and disabling the adopted low-pass filter gyro bias update function. The initial angle is aligned with the gold standard so the initial errors are zero. In the first static condition as shown in Figure 8a, the error increases linearly because the gyro bias is not removed. However, the error fluctuates drastically in dynamic stage. For example, in this test, the Euler angles error increase to 3.70°, 6.91° and 6.37° at 22 s, the errors start to fluctuate drastically in dynamic condition. When the gyro bias is removed properly, as shown in Figure 8b, the errors increase much slower. The errors were 1.76°, 0.52° and −1.72° even after 80 s. These results demonstrate the importance of gyro bias removal.

### 4.2. Test B: Pitch and Roll Estimations with Magnetic Disturbance 

**Figure 9:**
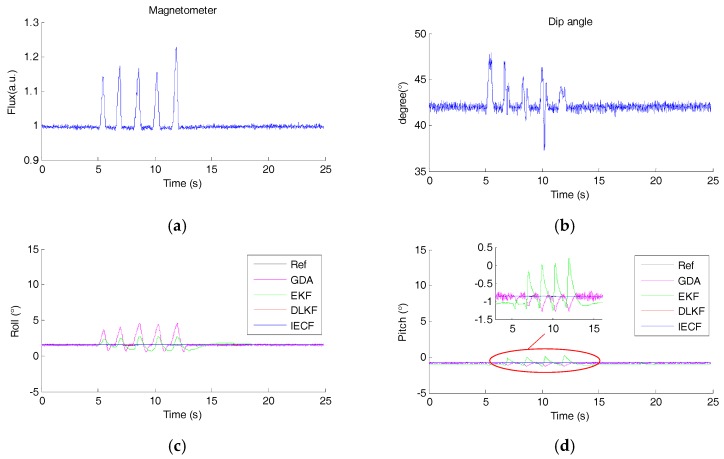

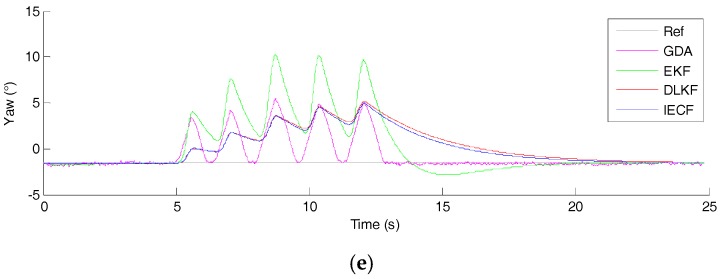
(**a**) The magnitude of the measured magnetic field. (**b**) The dip angle of the measured magnetic field. The estimated orientation of each sensor fusion methods, (**c**) Roll angle, (**d**) Pitch angle, (**e**) Yaw angle.

The magnitude and dip angle of the measured magnetic field are shown in Figure 9a,b. It can be seen that the magnetic disturbance occurred five times. The corresponding estimated Euler angles of different SFAs are plotted in Figure 9c–e. It is shown that for GDA and EKF, pitch and roll estimations are affected. The maximum roll deviations of GDA and EKF are 3.08° and 1.24°, pitch deviations are 0.41° and 1.20°, respectively. While for DLKF and IECF, pitch and roll estimations are nearly unchanged in the presence of the magnetic disturbance. This results agree with the theoretical analysis. In addition, the yaw estimations of all the algorithms are affected, the max errors of GDA, EKF, DLKF and IECF are 6.99°, 11.81°, 6.73° and 6.5°, respectively. This phenomena is reasonable because all magnetic disturbance compensation strategies are disabled.

### 4.3. Test C: Long-Time Stability of Yaw Angle with Continuous Magnetic Disturbance

**Figure 10:**
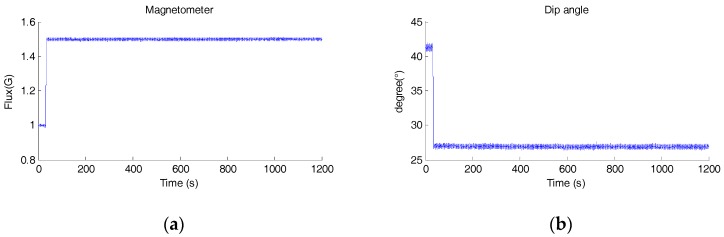
The measured magnetic field in the long time magnetic disturbance test, (**a**) magnitude of the magnetic field, (**b**) the dip angle of the magnetic field.

During the test, the magnitude and dip angle of the measured magnetic field is shown in Figure 10. Throughout the test, the MIMU was kept still for 20 min. A strong magnetic disturbance appeared at about 35 s and lasted to the end of the test. 

**Figure 11:**
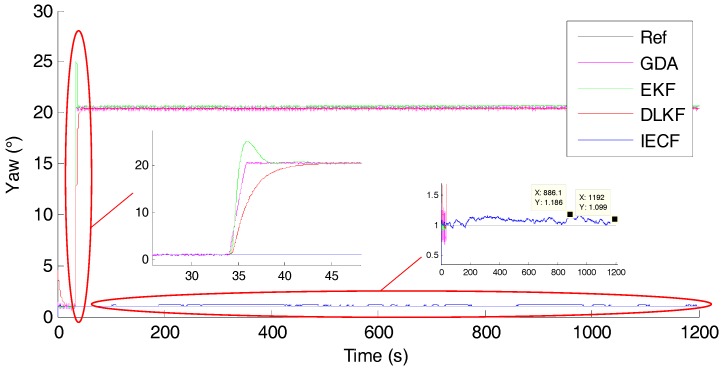
The estimated yaw angle of each sensor fusion method.

Yaw angle estimation is the main concern of this experiment. Figure 11 shows the estimated yaw angle of each sensor fusion method. 

It can be seen that yaw angles of GDA, EKF and DLKF diverge to the new wrong angle within 10 s after the introduction of the magnetic disturbance, yielding big yaw angle errors (>20°). The maximum deviations of GDA, EKF and DLKF are 21.44°, 21.66° and 21.45°, respectively. While the yaw angle estimation of IECF is nearly unaffected after the rejection of the magnetic disturbance. Throughout the test, the maximum deviation is 0.186°, and the deviation after 20 min is merely 0.099°. These results demonstrate that as long as the gyro bias is removed properly, the numerical integration of angular velocity in static condition is quiet reliable, and the rejection strategy of IECF is effective for strong continuous magnetic disturbance.

### 4.4. Test D: Yaw Estimation with Small Magnetic Disturbance in Static Condition

**Figure 12:**
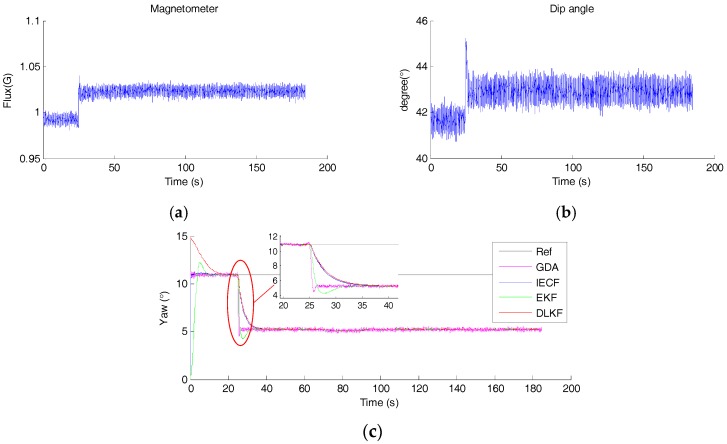
(**a**) The magnitude of the measured magnetic field, (**b**) The dip angle of the measured magnetic field, (**c**) The estimated yaw angle of each sensor fusion method.

Figure 12 shows the magnitude and dip angle of the measured magnetic field. It shows that a small magnetic disturbance is exerted to the MIMU after 25 s. The associated yaw angle estimation is shown in Figure 12c. It can be seen that for GDA, DLKF, EKF and IECF, yaw angles diverge to new magnetic field direction within 10 s, leading to errors. In this case, the errors for GDA, DLKF, EKF and IECF are 5.67°, 5.72°, 5.74° and 5.73°, respectively, and these errors depend on the direction of the new magnetic field. Besides, the convergence speeds of EKF and GDA are faster than DLKF and IECF. 

### 4.5. Test E: Dynamic Accuracy with Continuous Magnetic Disturbance 

**Figure 13:**
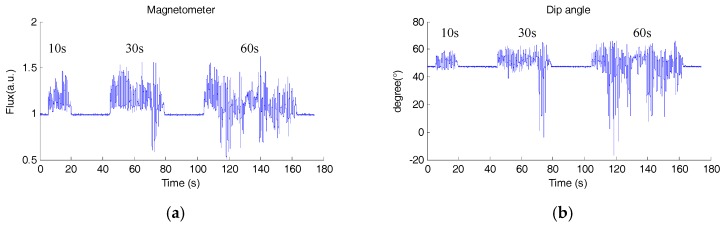
The magnitude (**a**) and the dip angle (**b**) of the measured magnetic field in dynamic accuracy test with continuous magnetic disturbance.

**Figure 14:**
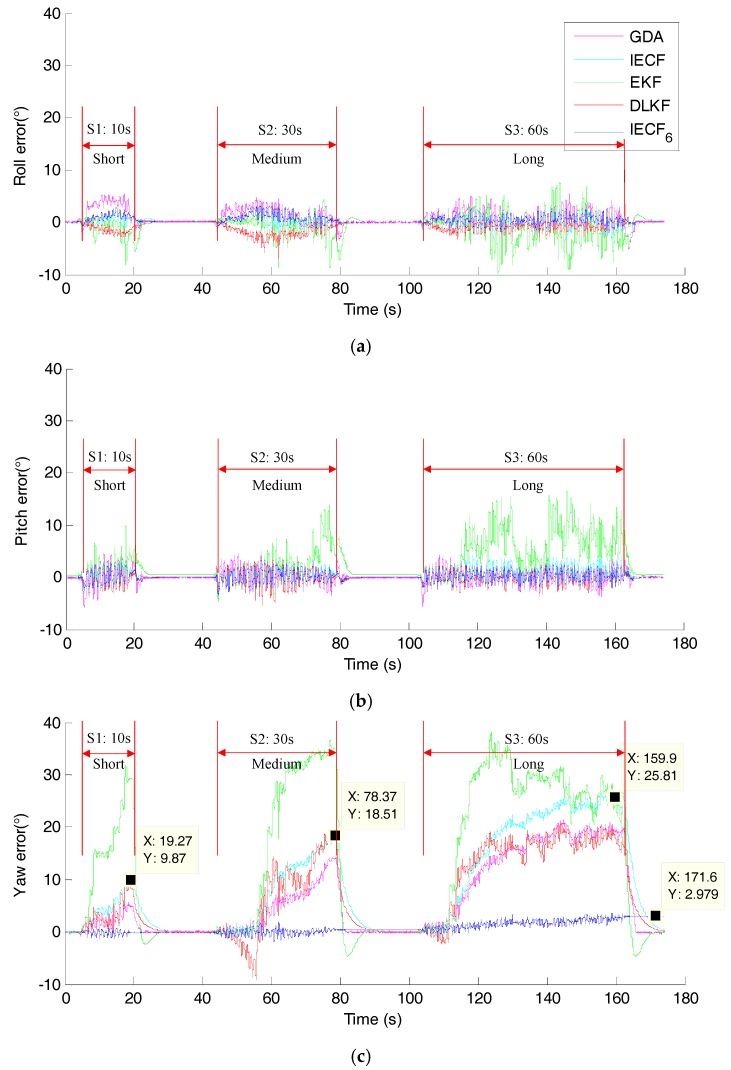
The error of orientation estimation of each sensor fusion methods. (**a**) Roll error, (**b**) Pitch error, (**c**) Yaw error.

Figure 13 shows the magnitude and dip angle of the measured magnetic field. It can be seen that there are three durations with continuous magnetic disturbance, i.e., 10 s (short), 30 s (medium) and 60 s (long). The orientation estimation errors for each sensor fusion method are shown in Figure 14, and the RMSEs of different SFAs are calculated and listed in Table 4. From the results, it can be seen that the attitude accuracies of EKF and GDA decreased under magnetic disturbance. On the whole, the RMS errors of yaw angle increase, as the increase of the duration of magnetic disturbance. The errors of all the four SFAs increase to more than 15° in 60 s disturbance tests, showing poor performance. However, the yaw error of IECF_6_ is much smaller than those of the selected SFAs, note that the initial yaw angle of IECF_6_ is aligned with the reference value.

## 5. Discussion

### 5.1. Sensor Fusion Methods Analysis

From the results of test A (Table 3), we can see that without magnetic disturbance, whether in static state and dynamic state, DLKF and IECF have similar performance, and EKF and GDA have similar performance. The performance of DLKF and IECF is better than that of EKF and GDA. A plausible explanation is that for DLKF and IECF, pitch and roll estimations are decoupled from magnetic reading. The variation of magnetic field does not affect the attitude performance of DLKF and IECF but it affects the EKF and GDA. In general, all of the SFAs show a good performance (in static conditions, all the errors are below 0.5°; in dynamic conditions, all the errors are below 1.5°). These results can be interpreted as in a slow motion state and a magnetically clean environment, the disturbance of acceleration and magnetic field are both low, and it is easy to calculate the orientation accurately. But when the magnetic disturbance are introduced, the differences between algorithms increase and adaptive strategies coping with magnetic disturbance become crucial.

The results of Test E (Figure 14) indicate that the simple threshold-based magnetic disturbance rejection strategy is not very effective, even when the duration of magnetic disturbance is short. And as the increase of the duration of the disturbance, the error increases. For the example of IECF, in 30 s and 60 s durations tests, the maximum errors increase to 12.61° and 17.68°, respectively. A possible interpretation is that the SFAs adopt some samples when the fluctuant magnitude is below the threshold. This situation may be improved when assuring that the magnitude is below the threshold for a certain amount of time. In addition, to clearly understand the performance of different sensor fusion methods under different conditions, a qualitative performance table is made as Table 5 based on the testing results. 

In some studies [6,7,16], researchers demonstrated the advantages of their proposed methods through comparison with the original GDA, an attitude filter with some well-known problems such as attitude estimation is not decoupled from magnetic reading [6,8,31,51]. In fact, the developer of GDA has realized the limitations of GDA and proposed a substituted new method, the IECF [40], which achieves a better performance. The IECF has the features of attitude estimation decoupled from magnetic reading and magnetic disturbance rejection. Therefore, it could be more appropriate to compare with the latest IECF instead of the GDA when proposing new SFAs.

The GDA and IECF have a common shortcoming that both of them have only one tuning parameter to determine the weighting factor of gyroscope. While the situation of external acceleration disturbance and magnetic disturbance may be different, sharing the same parameter may not compensate both magnetic disturbance and acceleration disturbance well. Separate parameters for them may provide better performance for a SFA. As for the selected EKF method. In test E, for the example of 10s short disturbances tests, the RMS errors of pitch and roll are 3.07° and 3.47°, respectively. Compared to 0.87° and 1.16° in Table 3 under dynamic conditions without magnetic disturbance, the errors are enlarged, which indicate that the pitch and roll estimations are seriously affected by magnetic disturbance. This phenomenon also demonstrate that pitch and roll estimations immunity to magnetic disturbance is important and should be considered as an essential feature for a robust SFA. In addition, another drawback of the EKF is that the reference direction of the earth’s magnetic field needs to be predefined, and inaccuracy in its definition will cause error in all of the three Euler angles.

Interestingly, in the condition of test E, with the alignment of the initial yaw angle, the IECF_6_ has the best performance. This is due to the fact that the yaw estimation of IECF_6_ merely relies on integration method. The maximum error is only 2.98° even after 160 s, which indicates the integration method is more reliable than fusing magnetometer data in the case of magnetic disturbance. The good performance of IECF_6_ manifests that the SFA still has room to improve. To achieve an equivalent performance as IECF_6_ but still to provide absolute yaw angle in the most challenging condition, an intelligent compensation strategy is desired.

Parameters of SFAs are factors that affect the performance of orientation estimation. In this paper, the parameters of all the SFAs were determined according to the original literature. The results in Figure 9e show that EKF and GDA have faster convergence speed. The results may be different for another set of parameters. However, the tuning of the parameters is out of the scope of this paper. The aim of this paper is not to draw conclusions about the differences among specifically parameterized versions of each method, but to understand the general features and differences among methods, in the aspects of pitch and roll estimations immunity to magnetic disturbances, the requirement of predefinition of geomagnetic field and etc. These features are irrelevant to parameters but critical to accurate orientation estimation in the presence of magnetic disturbance. The typical parameters used in each algorithm are sufficient to demonstrate the general features of the specific SFA. Moreover, the purpose of this paper is not to confirm which SFA is the best, and as shown from the results, no method can perform well enough under all the test conditions, and the popular SFAs still need further improvements. We hope the analysis in this paper could enable one to gain more insights into magnetic disturbance rejection strategies. 

### 5.2. The Key Considerations for Accurate Yaw Estimation in the Presence of Magnetic Disturbance

Magnetometer plays an important role in human motion analysis, and it is not only essential for removing heading drift but also to provide a common reference frame, in the condition of using multiple MIMUs [14]. Magnetometers are still useful even when the applications do not need an absolute heading angle but require a common reference frame. No magnetometer solution is only an option in methods that exploit kinematic constraints to handle orientation estimates from multiple sensors with different reference frames [52]. 

In the process of MIMU-based orientation estimation, the reference geomagnetic field is easily distorted by ambient ferromagnetic materials. When suffering from magnetic disturbance, estimating yaw angle through numerical integration of angular velocity is the only available option. If the yaw estimation based on integration can last longer, accurate yaw estimation against long-time magnetic disturbance is possible. The accuracy of the angular velocity is critical for the integration method, which mainly depends on constant bias, scaling factor, white noise, and in run bias stability [22]. The constant bias and scaling factor are determined during the calibration, and the in-run gyro bias is estimated online. In static condition, as long as the gyro bias is removed properly, it is easy to track the yaw angle accurately for a long time. As shown in Figure 11, the yaw angle of IECF is estimated by numerical integration when the magnetic disturbance is introduced. The deviation is still small (0.099°) even after 20 min, but in dynamic conditions, as shown in Figure 14c, the max deviation reach up to 2.98° in about 160 s, which is much bigger than in static conditions. The numerical integration is less reliable in dynamic condition. This is because the angular velocity error in a gyroscope is proportion to the reading, and the error grows bigger when angular velocity increases.

An important factor for accurate integration is the removal of gyro bias. The result in Figure 8 shows the importance of removal of gyro bias. The error fluctuates drastically in dynamic stage. This phenomenon can be interpreted as that for the well-accepted quaternion-based integration method [5,12], the errors of the previous quaternion will propagate to the predict quaternion with non-negligible errors, especially in the dynamic condition. This can be explained as following.

For the quaternion-based integration method, $q_{k+1\_ref}$ can be calculated by the expression $q_{k+1\_ref}=q_{\omega \_ref}⊗q_{k\_ref}$, where $q_{\omega \_ref}$ is the quaternion increment calculated by angular velocity integration, $q_{k+1\_ref}$ and $q_{k\_ref}$ are the (k + 1)th and kth estimated quaternion. ⊗ is quaternion multiplication. Supposing that $q_{e}$ is the quaternion error between the kth estimated quaternion $q_{k}$ and $q_{k\_ref}$, i.e., $q_{k}=q_{e}⊗q_{k\_ref}$, hence, $q_{k+1}=q_{\omega }⊗(q_{e}⊗q_{k\_ref}$). Consequently, the error between $q_{k+1}$ and $q_{k+1\_ref}$ is related to the error between $q_{\omega }⊗q_{e}$ and $q_{\omega \_ref}$. If $q_{e}$ is not a negligible error and $q_{\omega }$ is not close to unit quaternion. The propagation would cause a non-negligible error although the error between $\;q_{\omega }$ and $q_{\omega \_ref}$ is small. 

Another key component for accurate yaw estimation is the strategy dealing with magnetic disturbance. The SFA should be sensitive to magnetic disturbance, such that it can reject the distorted magnetic disturbance rapidly, preventing the increase of the orientation estimation error. However, sometimes small magnetic disturbances are hard to reject. A promising strategy for accurate yaw estimation is to rely more on gyroscope so that yaw angle diverges slower to new incorrect magnetic field direction. As shown in Figure 12c, if the convergence speed were lower, the increase of the error would be postponed. This action would contribute to improve the accuracy when encounter a fluctuated magnetic disturbance, which contain small magnetic disturbance certainly. In short, the key areas in improving the accuracy of yaw estimation are: (1) to reject the distorted magnetic disturbance as soon as possible; (2) to temporarily lower the convergence speed by adjusting the parameter when the magnetic disturbance is hard to reject.

### 5.3. Testing Method for Evaluating the Performance against Magnetic Disturbance

When evaluating the performance of a SFA, simple and standardized testing procedures should be considered. The testing conditions should contain challenge environments so that the strengths and weaknesses can be examined. However, in some existing studies [6,7,8,9,15,37] that focus on improving the performance against magnetic disturbance, the test conditions may not challenging enough based on the analysis in this paper. In most cases, only short duration magnetic disturbances, slow motion or static conditions, are considered to validate the proposed methods. Therefore, it is not clear how well these algorithms will perform in a more critical environment. The standardized testing procedures proposed in this paper can be used to objectively evaluate the performance against magnetic disturbance. The key features of a SFA against magnetic disturbance can be examined in the new procedures, including pitch and roll immunity to magnetic disturbance, gyro bias estimation and adaptive strategies for compensating the magnetic disturbance. Test E represents the most challenging condition mimicking the practical applications, because the 60 s magnetic disturbance is considered as long-duration magnetic disturbance in dynamic condition compared with the experiments in literature [6,9]. In this study, four commonly-used SFAs are submitted to the proposed standardized tests. Unfortunately, no one can perform well in all the test conditions. But through these complete tests, we have found what the weaknesses are and how to improve the existing methods. Further research should focus on proposing intelligent strategies which can adapt to different magnetic disturbance conditions, and achieve equivalent or better performance as IECF_6_ but still provide absolute yaw angle in seriously distorted environments.

## 6. Conclusions

In this paper, we focus on enabling a better understanding of how magnetic disturbances influence the attitude and heading in a MIMU-based orientation estimation algorithm, as well as the performance of current popular SFAs under standardized test conditions. To do so, we first reviewed four key components dealing with magnetic disturbance and analyzed the common methods used in each component. Then, we selected four representative sensor fusion methods to serve as a comparison study. To objectively evaluate the performance against magnetic disturbance of the selected sensor fusion methods, we propose a set of test scenarios including a series of extreme conditions. Experimental results expose the features of each method. For example, the pitch and roll estimations of GDA and EKF depend on magnetic reading; IECF and DLKF have better performance but still have room to improve. 

According to the analysis in this paper, a preferred SFA should have the following features: (1) on-line gyro bias compensation; (2) pitch and roll estimations immunity to magnetic disturbance; (3) adaptive strategies for magnetic disturbance. In addition, in the aspect of yaw accuracy, the preferred SFA had better perform as well as fusion algorithms that fuse accelerometer and gyroscope data, but still provide absolute yaw angles in a seriously magnetically distorted environment. The keys of accurate yaw estimation are to reject the distorted magnetic disturbance as soon as possible and to temporarily lower the convergence speed if the magnetic disturbance is hard to reject. Future work can focus on developing intelligent strategies for compensating the magnetic disturbance and then extending the findings to deal with acceleration distortions.

## Figures and Tables

**Table 1:** Optimally tuned parameter setting of each sensor fusion algorithms.

SFA	Parameter Settings	Other Features
DLKF [21]	$\sigma_{g}\;$= 0.001 rad/s $\sigma_{a}=0.2$ *g* $\sigma_{m}=0.2\;$a.u.	N/A
EKF [41]	$\sigma_{g}$ = 0.001 rad/s $\sigma_{a}=0.2$ *g* $\sigma_{m}=0.2\;$a.u.	N/A
GDA [12]	β=0.1	N/A
IECF [40]	K=0.5	Fast initialize. Pitch and roll estimations are decoupled from magnetic data.
IECF_6_ [40]	K=0.5	N/A

Note: Gyro standard deviation $\sigma_{g}$, rad/s. Accelerometer standard deviation $\sigma_{a}$, *g*. Magnetic sensor standard deviation $\sigma_{m}$, a.u.

**Table 2:** The standardized tests for performance evaluation under magnetic disturbance conditions.

Item	Objective	Test	Type
A	Static accuracy and dynamic accuracy	Static and dynamic accuracy without magnetic disturbance	General test
B	Attitude estimation dependency on magnetic disturbance	Pitch and roll estimation with magnetic disturbance	Special test
C	Gyro-bias estimation	Long-time stability of yaw angle with continuous magnetic disturbance	Special test
D	Convergence speed in small magnetically distorted condition	Yaw estimation with small magnetic disturbance in static condition	Special test
E	Dynamic accuracy in the most challenging condition	Dynamic accuracy with continuous magnetic disturbance	General test

**Table 3:** RMS errors and standard deviations of different sensor fusion methods under static and dynamic testing conditions, [mean ± SD, degree].

Test Condition	Euler Angles	DLKF	EKF	GDA	IECF
	Roll	0.16 ± 0.04	0.26 ± 0.03	0.29 ± 0.03	0.18 ± 0.04
Static	Pitch	0.15 ± 0.03	0.42 ± 0.02	0.30 ± 0.04	0.17 ± 0.04
	Yaw	0.13 ± 0.02	0.22 ± 0.05	0.25 ± 0.03	0.13 ± 0.01
	Roll	0.50 ± 0.14	0.87 ± 0.16	0.91 ± 0.13	0.53 ± 0.09
Dynamic	Pitch	0.52 ± 0.11	1.16 ± 0.22	1.00 ± 0.14	0.55 ± 0.10
	Yaw	0.40 ± 0.07	0.74 ± 0.14	0.68 ± 0.12	0.37 ± 0.09

**Table 4:** The RMS errors of the orientation estimations of different sensor fusion methods with continuous magnetic disturbance of different durations, [mean ± SD, degree].

Duration	Euler Angles	DLKF	EKF	GDA	IECF	IECF_6_
10 s Short	Roll	1.09 ± 0.28	3.07 ± 2.27	2.52 ± 0.67	1.11 ± 0.32	0.94 ± 0.25
	Pitch	1.07 ± 0.22	3.47 ± 1.59	1.58 ± 0.40	1.17 ± 0.18	1.03 ± 0.22
	Yaw	8.20 ± 3.22	16.94 ± 3.03	7.15 ± 3.51	6.87 ± 2.09	0.45 ± 0.09
30 s Medium	Roll	1.27 ± 0.54	2.73 ± 0.63	1.77 ± 0.28	0.97 ± 0.10	0.96 ± 0.19
	Pitch	1.10 ± 0.29	4.18 ± 0.91	1.32 ± 0.19	1.09 ± 0.17	0.92 ± 0.16
	Yaw	12.41 ± 2.21	23.49 ± 1.43	11.62 ± 3.48	12.61 ± 2.32	0.56 ± 0.30
60 s Long	Roll	1.12 ± 0.36	3.52 ± 0.99	1.74 ± 0.21	1.10 ± 0.16	0.96 ± 0.19
	Pitch	0.98 ± 0.16	5.24 ± 1.13	1.23 ± 0.09	1.07 ± 0.24	0.89 ± 0.12
	Yaw	16.51 ± 1.63	25.90 ± 2.80	16.79 ± 1.39	17.68 ± 1.78	1.02 ± 0.59

**Table 5:** The qualitative performance of different sensor fusion methods.

Conditions	GDA	DLKF	EKF	IECF	IECF_6_
Test A	Good	Good	Good	Good	-
Test B	Poor	Good	Poor	Good	-
Test C	-	-	-	Good	-
Test D	Poor	Poor	Poor	Poor	-
Test E-10s	Poor	Poor	Poor	Poor	Good
Test E-30s	Poor	Poor	Poor	Poor	Good
Test E-60s	Poor	Poor	Poor	Poor	Good

Note: For easy understanding, the performance of each SFAs is qualitative with the standard: Good (≤2°), Acceptable (2°–5°), Poor (>5°), “-” means it make no sense to qualify the performance.
